# Variation of Expression Levels of Seven Housekeeping Genes at Different Life-History Stages in *Porphyra yezoensis*

**DOI:** 10.1371/journal.pone.0060740

**Published:** 2013-04-18

**Authors:** Xiaojie Wu, Aiyou Huang, Meiling Xu, Chao Wang, Zhaojun Jia, Guangce Wang, Jianfeng Niu

**Affiliations:** 1 College of Marine Science and Engineering, Tianjin University of Science and Technology, Tianjin, China; 2 Institute of Oceanology, Chinese Academy of Sciences, Qingdao, China; 3 Graduate School, Chinese Academy of Sciences, Beijing, China; Rutgers University, United States of America

## Abstract

In order to identify the optimal internal control for relative real-time PCR when studying target gene expression in the red alga *Porphyra yezoensis*, we quantified the expression of seven housekeeping genes (18S ribosomal RNA, 30S ribosomal protein S8, Polyubiquitin-2, Glyceraldehyde-3-phosphate dehydrogenase, Elongation factor 1-alpha, Beta-tubulin and Actin 3) at different life-history stages. Absolute quantification was done by normalization to total RNA quantity and by normalization to genomic DNA quantity. We used these two normalization approaches, comparing the differences of expression levels of all candidate housekeeping genes between any two generations and across three life-history stages (filamentous sporophytes, leafy gametophytes and conchospores). We found *GAPDH* had the best stability in all cases and we recommend that *GAPDH* be considered as a potential internal control for gene expression studies at different life-history stages in *P. yezoensis*.

## Introduction

*Porphyra* (Bangiales, Rhodophyta) includes more than 100 species distributed worldwide [1]. Owing to its economic importance and health benefits [2]–[4], *Porphyra* is treated as essential material in practical application research [5], [6]. *Porphyra* grows on intertidal marine rocks, surviving under extreme conditions such as high temperature, strong light and desiccation for many hours each day [7] and is, therefore, suitable experimental material for understanding how intertidal organisms respond to various forms of stress [8], [9]. Moreover, the multiphasic life history of *Porphyra* can be observed in the laboratory within a few months [10], accelerating research on growth, reproduction and photosynthetic mechanisms [11]–[13]. These characteristics have prompted increasing attention to *Porphyra,* which has been proposed as a model system for physiological and genetic investigation [14], [15].

The red alga *Porphyra yezoensis* is one of the most extensively cultivated species in Japan, China and South Korea. It has a complex heteromorphic life cycle with an alternation between macroscopic haploid leafy gametophytes and microscopic filamentous sporophytes. Shell-boring filamentous sporophytes release conchospores that can germinate and grow into leafy gametophytes [14], [16], [17]. It is apparent that different life-history stages of *P. yezoensis* exhibit different morphological traits and occur under distinct growth conditions; the gametophytes live freely in seawater and the sporophytes penetrate the shells. One question that needs to be answered is whether expression levels of housekeeping genes are constant in the various life-history stages?

The analysis of target gene expression at different life-history stages is an increasingly significant field of life science research [11], [18], [19]. In order to conduct a gene expression assay it is crucial to choose a suitable internal control gene that is expressed stably and, thus, the selection of this internal control gene is an absolute prerequisite for accurate quantification of target gene expression [20]–[22]. In fact, it has become clear that there is no gene that can be used as internal control for all species [23]–[26]. However, no ideal internal control for gene expression analysis in *P. yezoensis* has been identified. In addition, it is worth noting that results obtained by normalization to a single internal control are generally less rigorous or even inaccurate, and that results obtained by normalization to two or more different internal controls are contradictory in some cases. Owing to the complex life-history of *P. yezoensis*, this problem is particularly pertinent to the investigation of gene expression. The requirement for suitable internal control genes for normalization is increasingly stringent for in-depth research into *P. yezoensis*, for which the mechanism underlying gene regulation and expression across the entire life-history is extremely important.

Our objective was to identify internal control genes suitable for the expression profile of a target at different life-history stages. We chose seven housekeeping genes commonly used as likely candidates and the transcript numbers of these genes were determined with absolute quantification. The results given here serve as a reliable reference for selection of internal controls in future experimental designs.

## Materials and Methods

### *P. yezoensis* culture

The filamentous sporophytes were cultured in sterile seawater with added nutrients with constant aeration at 20°C with 50°μmol of photons m^−2^ s^−1^ (12 h light/12 h dark). The nutrient-enriched seawater was changed every week. Basically, the shell-boring sporophytes were incubated in fresh filtered seawater at 24°C with PES medium under 20 μmol of photons m^−2^ s^−1^ with a 12 h light/12 h dark cycle. After a few days, the temperature was reduced gradually to 20°C and we started to collect conchospores from the shells. Before collection, the shells were brushed with filtered seawater to remove any contamination. The seawater containing conchospores was poured slowly into a glass beaker, then transferred to a 1.5 ml microcentrifuge tube and centrifuged (Eppendorf Centrifuge 5804R; Eppendorf AG, Hamburg, Germany) at 4000 *g* for 5 min at room temperature [27]. The conchospores that had already been isolated were immediately frozen in liquid nitrogen and stored at –80°C. The leafy gametophytes were cultivated at 15°C with 50 μmol of photons m^−2^ s^−1^ in 12 h light/12 h dark cycle.

### RNA extraction and cDNA synthesis

Total RNA was extracted from samples at different life-history stages using a Tiangen RNAprep pure plant kit (Tiangen, Beijing) according to the manufacturer's instructions. It should be noted that RNA extractions from sporophytes and gametophytes were replicated three times. However, although collected every day, the amount of conchospores was insufficient to complete replicate experiments. For this reason, RNA extraction from conchospores was conducted only one time.

Before the extraction of RNA, each sample was dried with sterile filter paper to avoid degradation, ground to a powder in liquid nitrogen and then weighed. Purified RNA was eluted with RNase-free water and immediately stored at –80°C. RNA integrity was checked by electrophoresis in a 2% (w/v) agarose gel, RNA purity was estimated by measuring OD 260/280 absorbance ratio, and the concentration of RNA was determined with a Qubit® Fluorometer and the corresponding RNA Assay Kit (Invitrogen, USA). RNA yield was calculated as RNA quantity/sample weight. RNase-free DNase I (Promega, USA) was used to eliminate genomic DNA contamination before RT-PCR. First-strand cDNA was synthesized using M-MLV reverse transcriptase (Promega, USA) with random primers on a PCR apparatus (Eppendorf, Genman) following the protocols suggested by the manufacturer. The cDNA product was stored at –20°C.

### Genomic DNA extraction

Genomic DNA was extracted from samples at different life-history stages using the Universal Genomic DNA Extraction Kit (TaKaRa Biotechnology, Dalian, China) according to the manufacturer's instructions. Similarly, DNA extractions from sporophytes and gametophytes were replicated three times, and DNA extraction from conchospores was conducted only one time. In order to avoid error caused by grinding, samples of the same powder were used for extraction of RNA and DNA. In addition, the amount of materials used to extract RNA and DNA also was the same in terms of weight. DNA quality was evaluated by measuring OD 260/280 absorbance ratio. The concentration of genomic DNA was determined with a Qubit® Fluorometer and the corresponding DNA Assay Kit (Invitrogen, USA). DNA yield was calculated as DNA quantity/sample weight.

### Primer design

The sequences of commonly used housekeeping genes 1*8S, RPS8, PUB-2, GAPDH, EF1alpha, TubB* and *Act3* were downloaded from the Genbank database (http://www.ncbi.nlm.nih.gov). Primer design and evaluation were completed with Primer Premier 5.0 (Premier Biosoft International, Palo Alto, CA) and Oligo Primer analysis software (Wojciech and Piotr Rychlik Copyright, version 6.31). The criteria used for primer design included a primer length of 17–21 bp, a PCR product size of 104–187 bp and other general rules (Table 1).

**Table 1:** Primer names, sequences and PCR product size of selected candidate housekeeping genes.

Gene name	Gene ID	Primer name	Primer sequence 5′→3′	Product size (bp)
18S Rrna (*18S*)	108742709	18S-S	CGACCGTTTACTGTGAAG	160
		18S-A	GACAATGAAATACGAATGCC	
Actin 3 (*Act3*)	204022115	ACT3-S	CCAAGCAGAAGGGCATCAT	173
		ACT3-A	CCGCAGCTCCGAGTAGAA	
Elongation factor 1-alpha (*EF1alpha*)	3978990	EF1a-S	GTCACCAGGCATAACCAT	135
		EF1a-A	GAGGGCGGAAGACATACT	
Glyceraldehyde-3-phosphate dehydrogenase (*GAPDH*)	168274242	GAPDH-S	CCAACAAGTGGGAGTAAGCG	104
		GAPDH-A	GGACAGAACCGAACAGCGTA	
Polyubiquitin-2 (*PUB-2*)	114159827	PUB-S	ATTCACGCAACACGCACTT	116
		PUB-A	ACGGGGAGCGGTGAGAT	
30S ribosomal protein S8 (*RPS8*)	116266137	RPS8-S	CTGGGCAACTCTTTATGAT	143
		RPS8-A	GAACAAATGGGTGAAGGTAT	
Beta-tubulin (*TubB*)	56384661	TubB-S	CGGCATTTATCGGCAACT	126
		TubB-A	CCATCTCGTCCATTCCCTC	

### Construction of DNA standards

The fragment of each housekeeping gene was amplified by PCR, using the specific primers (Table 2). PCR amplicon standard was purified using the E.Z.N.A TM Gel Extraction Kit (OMEGA Bio-Tek, Doraville, GA, USA) and then quantified with a Qubit® fluorometer and the corresponding dsDNA HS Assay Kit (Invitrogen Crop., Carlsbad, CA, USA). Transcript number was calculated as:
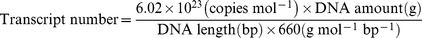
(1)


**Table 2:** Specific primers used for amplifying PCR amplicon standards.

Gene	Primer name	Primer sequence 5′→3′	Amplicon size (bp)
*18S*	18S-SR	TGCCTTGTTGCCAGTGGGAGTT	878
	18S-AR	TTACCCGAGCCTTCCGACCC	
*Act3*	ACT3-SR	CCAAGCAGAAGGGCATCAT	813
	ACT3-AR	TGCCCGCAAACATCGTCGTG	
*EF1alpha*	EF1a-SR	CTACGGTACGGCCACCTTCTC	871
	EF1a-AR	GACGGTCCAATGCCACAAACT	
*GAPDH*	GAPDH-SR	ACGCCACGGACGACATT	675
	GAPDH-AR	CGGACAGAACCGAACAGC	
*PUB-2*	PUB-SR	ATTCACGCAACACGCACTT	1523
	PUB-AR	CCTCACAAGAGTCACATCCC	
*RPS8*	RPS8-SR	TCACCACCAAGACCATCA	344
	RPS8-AR	CGTATTCGTAACGCAAACT	
*TubB*	TubB-SR	TACAACTGCACGCTTTCGG	781
	TubB-AR	CGCCTTCGGCATACTCG	

### Quantification of the tested genes in various samples

Real-time PCR was done with a BIO-RAD IQ5 real-time PCR detection system (Bio-Rad, USA) containing 9 μl of 2× SYBR® Green Master Mix (Tiangen, Beijing), 2 μl of template (standard diluted in series or cDNA diluted 10-fold), 5 μl of sense and antisense primer (2 μM) and made to a final volume of 20 μl with RNase-free water. Conditions were: 3 min at 94°C, followed by 40 cycles of 15 s at 94°C, 40 s at 59°C and 30 s at 72°C. The melting curve was generated by heating for 30 s from 55°C to 95°C, with a ramp speed of 0.5°C/cycle. A 10-fold series dilution of the standard was used to construct the standard curves [28]–[30] by plotting the logarithm of the threshold cycles against the logarithm of the transcript number of the template. The levels of all unknown samples should be distributed within the range of the standard curve. Each sample was amplified in triplicate. Amplification efficiency (*E*) and the linear correlation coefficient (*R* ^2^) were used to evaluate primer efficiency:
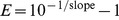
(2)


On the basis of the *C*_t_ values obtained, the transcript numbers of all tested genes in different samples were calculated and normalized to total RNA quantity and to genomic DNA quantity. The expression stability of all candidate housekeeping genes was evaluated by comparing the differences of observed transcript numbers between any two generations and across three life-history stages [31]. The entire protocol, from nucleic acid extraction to Real-time PCR, was replicated.

## Results

### Quality and yields of total RNA and genomic DNA

An OD 260/280 ratio of 1.8–2.0 is usually considered as an acceptable indicator of good nucleic acid quality. In this study, OD 260/280 ratios of total RNA and genomic DNA extracted from different samples varied between 1.8 and 2.0 (Table 3).

**Table 3:** The OD 260/280 ratios of extracted nucleic acid.

	Sporophytes	Gametophytes	Conchospores
RNA	1.88–2.04	1.92–1.99	1.85
DNA	1.90–1.96	1.94–2.01	1.92

**Figure 1:**
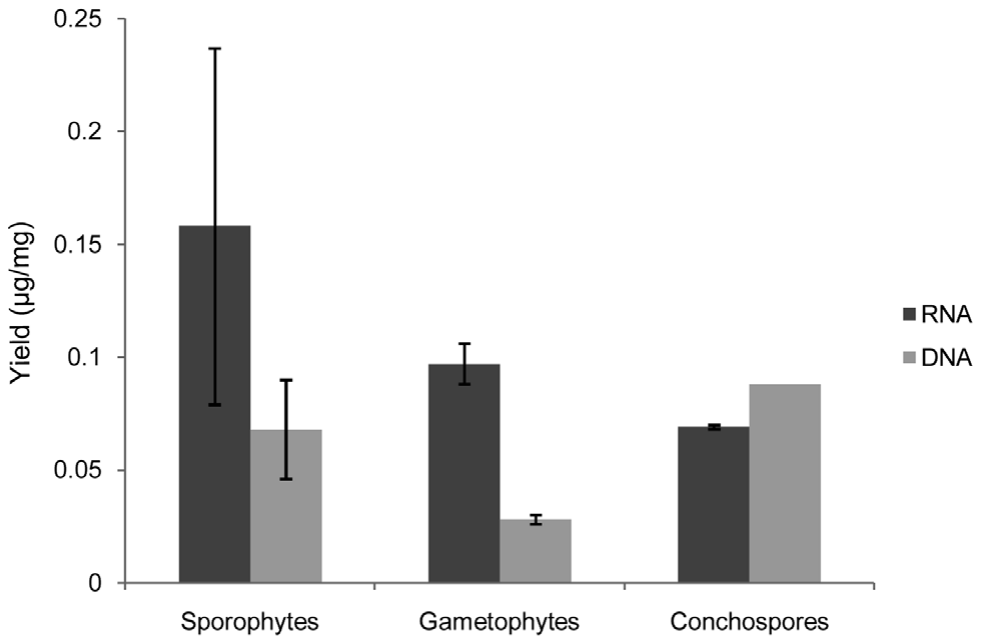
Yields of total RNA and genomic DNA of various sample groups.

The yields of total RNA and genomic DNA were calculated by dividing quantity by the corresponding sample weight. The mean RNA yield was highest in the sporophytes (0.158±0.059 µg/mg) and lowest in the conchospores (0.069 µg/mg), whereas the mean DNA yield was highest in the conchospores (0.088 µg/mg) and lowest in the gametophytes (0.028±0.002 µg/mg) (Figure 1).

### Primers specificity and efficiency

**Figure 2:**
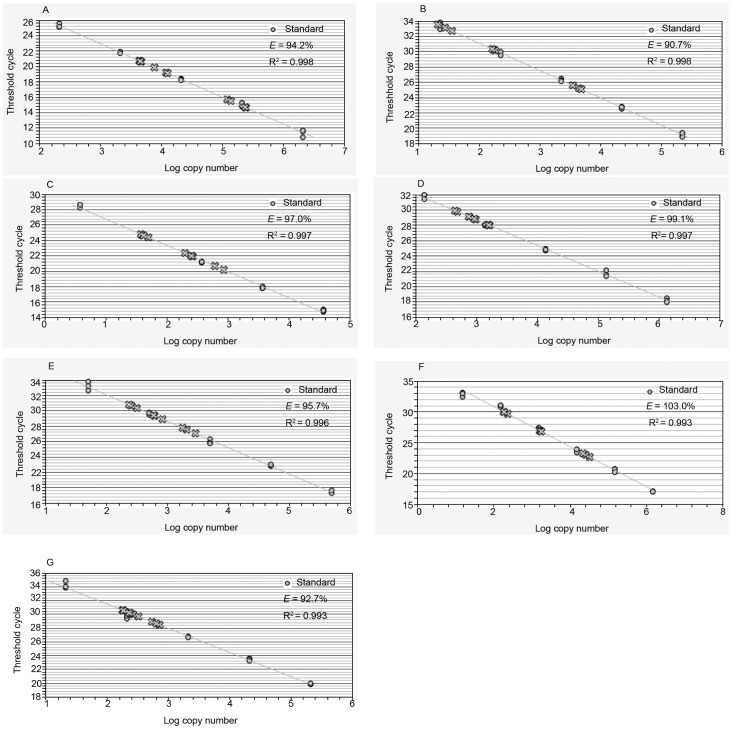
The standard curves constructed for *18S* (A), *Act3* (B), *EF1alpha* (C), *GAPDH* (D), *PUB-2* (E), *RPS8* (F), *TubB* (G). The results showed that amplification efficiency was between 96% and 103%, and linear correlation coefficient was >0.99.

**Figure 3:**
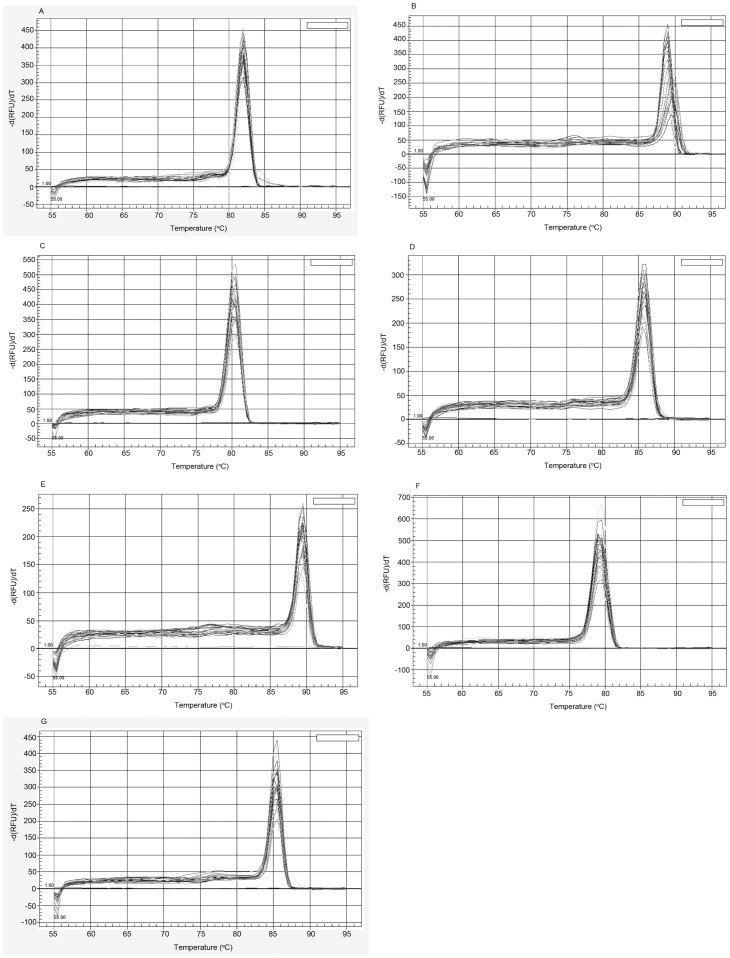
The melting curve analysis for *18S* (A), *Act3* (B), *EF1alpha* (C), *GAPDH* (D), *PUB-2* (E), *RPS8* (F), *TubB* (G). Melting peaks were examined with standard samples and unkown samples (sporophytes, gametophytes and conchospores). The melting curve for each gene had only one peak.

In this study, the amplification efficiency of all PCRs was between 90% and 105%, and *R* ^2^ was >0.980 (Figure 2). In the melting curve analysis, the amplicons of all seven genes revealed a single product with a melting temperature in accord with the expected value (Figure 3).

### Expression profiling and stability of candidate housekeeping genes: normalization to total RNA

On the basis of standard curves, the transcript number of the housekeeping gene in different samples was obtained by normalizing to total RNA. The expression levels of the genes were *18S* > *GAPDH* > *RPS8* > > *EF1alpha* > *PUB-2*> *TubB* > *Act3*, range 2.25×10^3^–3.47×10^7^ (based on the expression level in the sporophytes), and the expression of 18S was the highest at all life-history stages. The trend in expression levels was the same for all candidate housekeeping genes at different life-history stages: conchospores > sporophytes > gametophytes (Table 4).

**Table 4:** Transcript numbers of candidate housekeeping genes in *P. yezoensis* determined by absolute quantitative analysis normalized to total RNA quantity (copies/μg).

Gene	Sporophytes		Gametophytes		Conchospores	
	Mean	Std	Mean	Std	Mean	Std
*18S*	3.47×10^7^	1.15×10^6^	1.11×10^7^	7.91×10^5^	7.77×10^9^	1.58×10^8^
*Act3*	2.25×10^3^	2.81×10^2^	9.70×10^2^	1.12×10^2^	2.18×10^5^	3.13×10^4^
*EF1alpha*	5.51×10^4^	6.16×10^3^	1.69×10^4^	9.39×10^2^	4.03×10^6^	6.19×10^5^
*GAPDH*	6.36×10^4^	7.54×10^3^	5.82×10^4^	1.22×10^4^	1.29×10^5^	6.74×10^3^
*PUB-2*	1.17×10^4^	3.26×10^3^	1.45×10^3^	9.29×10^1^	1.32×10^5^	4.67×10^4^
*RPS8*	6.19×10^4^	5.15×10^3^	1.52×10^4^	1.51×10^3^	4.70×10^6^	3.06×10^5^
*TubB*	3.12×10^3^	5.59×10^2^	1.43×10^3^	3.30×10^2^	3.07×10^4^	4.03×10^3^

We ranked the tested genes by the difference between the smallest and the largest transcript number of each gene across all samples (Table 5). *GAPDH* had the smallest difference (2.2-fold), and there was little variation across all samples. The difference for *18* *S* varied greatly, ranging from 1.11×10^7^ to7.77×10^9^ copies. *Act3, EF1alpha* and *RPS8* showed huge variance with >100-fold differences. The difference was 21.5-fold for *TubB* and 91.1-fold for *PUB-2*.

**Table 5:** Stability of candidate housekeeping gene expression *in P. yezoensis* (from smallest to largest difference) determined by difference across all samples.

Rank	Gene	Difference (RNA)	Gene	Difference (DNA)
1	*GAPDH*	2.2 (C/G)	*GAPDH*	2.0 (G/C)
2	*TubB*	21.5 (C/G)	*TubB*	4.9 (C/G)
3	*PUB-2*	91.1 (C/G)	*PUB-2*	20.6 (C/G)
4	*Act3*	225.2 (C/G)	*EF1alpha*	54.1 (C/G)
5	*EF1alpha*	238.9 (C/G)	*Act3*	51.0 (C/G)
6	*RPS8*	310.1 (C/G)	*PRS8*	70.2 (C/G)
7	*18S*	700.6 (C/G)	*18S*	158.6 (C/G)

Results are calculated as maximum/minimum. Difference (RNA) is determined by transcript number normalized to total RNA quantity and Difference (DNA) is determined by transcript number normalized to genomic DNA quantity.

We calculated the differences of expression values between any two generations. The differences between the expression levels of all tested genes in the sporophytes and in the gametophytes were relatively small, ranging from 1.1-fold for *GAPDH* to 8.1-fold for PUB-2 (Table 6). According to the difference between the sporophytes and the conchospores, we found that *GAPDH* was still the most satisfactory of the tested genes; values for the other tested genes ranged from 9.9-fold to 224.0-fold (Table 7). Ranking by the differences between the gametophytes and the conchospores was identical with that based on differences across all samples (Table 8).

**Table 6:** Stability of candidate housekeeping gene expression *in P. yezoensis* (from smallest to largest difference) determined by difference between the sporophytes and the gametophytes.

Rank	Gene	Difference (RNA)	Gene	Difference (DNA)
1	*GAPDH*	1.1 (S/G)	*GAPDH*	1.4 (G/S)
2	*TubB*	2.2 (S/G)	*TubB*	1.5 (S/G)
3	*Act3*	2.3 (S/G)	*Act3*	1.7 (S/G)
4	*18S*	3.1 (S/G)	*18S*	2.1 (S/G)
5	*EF1alpha*	3.3 (S/G)	*EF1alpha*	2.2 (S/G)
6	*RPS8*	4.1 (S/G)	*RPS8*	2.7 (S/G)
7	*PUB-2*	8.1 (S/G)	*PUB-2*	5.4 (S/G)

Results are calculated as maximum/minimum. Difference (RNA) is determined by transcript number normalized to total RNA quantity and Difference (DNA) is determined by transcript number normalized to genomic DNA quantity.

**Table 7:** Stability of candidate housekeeping gene expression *in P. yezoensis* (from smallest to largest difference) determined by difference between the sporophytes and the conchospores.

Rank	Gene	Difference (RNA)	Gene	Difference (DNA)
1	*GAPDH*	2.0 (C/S)	*GAPDH*	1.5 (S/C)
2	*TubB*	9.9 (C/S)	*TubB*	3.3 (C/S)
3	*PUB-2*	11.2 (C/S)	*PUB-2*	3.8 (C/S)
4	*EF1alpha*	73.1 (C/S)	*EF1alpha*	24.7 (C/S)
5	*RPS8*	76.0 (C/S)	*RPS8*	25.6 (C/S)
6	*Act3*	97.1 (C/S)	*Act3*	29.2 (C/S)
7	*18S*	224.0 (C/S)	*18S*	75.6 (C/S)

Results are calculated as maximum/minimum. Difference (RNA) is determined by transcript number normalized to total RNA quantity and Difference (DNA) is determined by transcript number normalized to genomic DNA quantity.

**Table 8:** Stability of candidate housekeeping gene expression *in P. yezoensis* (from smallest to largest difference) determined by difference between the gametophytes and the conchospores.

Rank	Gene	Difference (RNA)	Gene	Difference (DNA)
1	*GAPDH*	2.2 (C/G)	*GAPDH*	2.0 (G/C)
2	*TubB*	21.5 (C/G)	*TubB*	4.9 (C/G)
3	*PUB-2*	91.1 (C/G)	*PUB-2*	20.6 (C/G)
4	*Act3*	225.2 (C/G)	*Act3*	51.0 (C/G)
5	*EF1alpha*	238.9 (C/G)	*EF1alpha*	54.1 (C/G)
6	*RPS8*	310.1 (C/G)	*RPS8*	70.2 (C/G)
7	*18S*	700.6 (C/G)	*18S*	158.6 (C/G)

Results are calculated as maximum/minimum. Difference (RNA) is determined by transcript number normalized to total RNA quantity and Difference (DNA) is determined by transcript number normalized to genomic DNA quantity.

### Expression profiling and stability of candidate housekeeping genes: normalization to genomic DNA

The expression levels of the tested genes were *18S* > GAPDH > *RPS8* > > *EF1alpha* > *PUB-2*> *TubB* > *Act3*, ranging from 5.86×10^3^ to 8.06×10^7^ (based on the expression level in the sporophytes). The transcript abundance of all the tested genes was greatest for the conchospores, except *GAPDH*, which was expressed highest in the gametophytes (Table 9).

**Table 9:** Transcript numbers of candidate housekeeping genes in *P. yezoensis* determined by absolute quantitative analysis normalized to genomic DNA quantity (copies/μg).

Gene	Sporophytes		Gametophytes		Conchospores	
	Mean	Std	Mean	Std	Mean	Std
*18S*	8.06×10^7^	2.67×10^6^	3.84×10^7^	2.74×10^6^	6.09×10^9^	1.24×10^8^
*Act3*	5.86×10^3^	6.53×10^2^	3.36×10^3^	3.86×10^2^	1.71×10^5^	2.45×10^4^
*EF1alpha*	1.28×10^5^	1.43×10^4^	5.84×10^4^	3.25×10^3^	3.16×10^6^	4.85×10^5^
*GAPDH*	1.48×10^5^	1.75×10^4^	2.02×10^5^	4.22×10^4^	1.01×10^5^	5.29×10^3^
*PUB-2*	2.72×10^4^	7.58×10^3^	5.01×10^3^	3.22×10^2^	1.03×10^5^	3.66×10^4^
*RPS8*	1.44×10^5^	1.20×10^4^	5.25×10^4^	5.23×10^3^	3.68×10^6^	2.40×10^5^
*TubB*	7.25×10^3^	1.30×10^3^	4.94×10^3^	1.14×10^3^	2.41×10^4^	3.16×10^3^

We compared the difference of expression levels of the tested genes between any two generations and across three life-history stages. The expression levels of all the tested genes were similar between the sporophytes and the gametophytes, ranging from 1.4-fold to 5.4-fold (Table 6). By comparing the transcript profile of sporophytes or gametophytes against conconspores and analyzing the difference of transcript numbers across three life-history stages, we found that *18S*, *Act3*, *EF1alpha*, *PUB-2*, *RPS8* and *TubB* were not good choices as internal controls in these cases. However, GAPDH displayed the greatest level of stability (Table 5, Table 7, Table 8).

## Discussion

It is essential to identify an appropriate internal control for accurate analysis of gene expression at different life-history stages and in various tissues [32], [33]. At present, 18S ribosomal RNA, glyceraldehyde-3-phosphate dehydrogenase and β-actin are most commonly selected as internal controls for higher animals and plants [34]–[38]. Other housekeeping genes, such as Elongation factor and 30S Ribosomal Protein Subunit, have been proposed to be suitable for normalization in real-time PCR experiments under certain conditions [23], [38]. Generally, in such studies, differences in gene expression have been compared in different organs and tissues or under various experimental conditions but at the life-history stage had the same ploidy. Although expression profiling can be done by normalizing against the above-mentioned internal control genes, there are still researchers advocating the use of two or more internal control genes, in order to ensure accuracy [21], [39], [40]. Thus, for *P. yezoensis* with a complex life history involving changes in ploidy, it is necessary to choose optimal internal controls for gene expression studies with caution.

Absolute quantification requires standardization, and several approaches have been suggested [30], [41]. Ideally, the gene transcript number is standardized to the number of cells [21], [28]; however, *P. yezoensis* is a multicellular organism and accurate enumeration of cells is impossible. A strategy based on total RNA mass quantity is widely used [42]–[44] and we suggest normalization against the genomic DNA quantity of tested samples could be used. In order to identify a suitable internal control for *P. yezoensis*, we have quantified the expression of seven housekeeping genes at different life-history stages with absolute quantification by normalization to total RNA quantity and to genomic DNA quantity.

Besides, absolute quantification requires the construction of a standard curve for each target gene, using DNA standard. Although it had been demonstrated that the amplification efficiency was unchanged by using circular or linear DNA as the standard [29], the types of DNA standard (circular plasmid, linearized plasmid and linear PCR amplicon) affected quantification accuracy. A recent study showed that a circular plasmid was unsuitable as a standard, by which gene transcript number was overestimated seriously, whereas the linear standards gave highly accurate estimates [45]. In this study, a linear PCR amplicon was used as the standard for constructing standard curve due to its high flexibility and sensitivity. An ideally suited internal control gene is one that shows no, or only a limited, variation of expression across the sample set [46]. Consequently, we chose the relatively stably expressed gene *GAPDH* as a suitable internal control for relative quantification under certain experimental designs. No matter which normalization method was applied, when differences of gene expression levels between any two generations or across three generations were compared, the ranking of housekeeping genes was consistent, identifying *GAPDH* as the most stable of the tested genes with differences ranging from 1.4- fold to 2.0-fold. However, the expression levels of the other six tested genes varied greatly and to different degrees. Based on the above results, we believed it was appropriate to use *GAPDH* as the internal control in this study.

It should be pointed out, however, that the results showed enormous expression differences exist in housekeeping genes between vegetative cells and conchospores. Released conchospores are looking for matrix to attach rapidly by amoeboid movement. In amoeboid movement, rich expression of actin is significant for cytoskeletal polymerization and depolymerization. Simultaneously, ribosome and some related functional proteins are synthesized abundantly to meet the need of the movement. Therefore, it is understandable that there is large expression differences between vegetative and conchospores.

In conclusion, we recommend that *GAPDH* be used as the internal control for gene expression studies at different life-history stages of *P. yezoensis*, allowing accurate quantification of target gene expression by real-time quantitative PCR experimental designs.
